# Supplementary material for: Roles of the membrane-binding motif and the C-terminal domain of RNase E in localization and diffusion in E. coli
Source: eLife. 2025 Nov 7;14:RP105062. doi: 10.7554/eLife.105062 (PMC12594526; doi:10.7554/eLife.105062)
Supplement: Supplementary file 1. [file elife-105062-supp1.pdf]

Supplementary file 1. List of strains used in this study

Strain number	Genotype	Source	Use
SK1	MG1655		Cloning<br>Doubling time
SK47	BW25993 *rplA::rplA-mEos2*	(Sanamrad et al., 2014)	Imaging
SK52	MG1655 Δ*araFGH araE*::P13-*araE*	C. Jacobs-Wagner	Cloning
SK72	NCM3416 *rne::rne-mCherry* FRT-*cat*-FRT	(Strahl et al., 2015)	Cloning
SK98	MG1655 Δ*lacYA*	(Kim et al., 2019)	qRT-PCR<br>Cloning
SK105	MG1655 Δ*lacIZYA*	CJW6643(Kim et al., 2019)	Cloning
SK107	MG1655 *rne::rne*(ΔMTS)-*mCherry*	CJW5685(Thappeta et al., 2024)	Cloning
SK186	MG1655 *rne::rne*(1-592)-*yfp* FRT-*kan*-FRT	C. Jacobs-Wagner, JRH474	Cloning
SK187	MG1655 *rne::rne-mEos3.2* FRT-*kan*-FRT	C. Jacobs-Wagner, JRH475	Imaging
SK213	BW25113 *hupA::hupA-mcherry* FRT-*kan*-FRT	CJW5158 (Xiang et al., 2021)	Cloning
SK249	MG1655 *rne::rne*ΔMTS-*mEos3.2* FRT-*kan*-FRT	This study	Imaging
SK290	MG1655 *rne::rne-mEOS3.2*	This study	Cloning
SK292	MG1655 *lacYA::lacY-mEos3.2* FRT-*kan*-FRT	This study	Imaging
SK304	MG1655 *rne::rne-mEos3.2, ΔrhlB::*FRT-*kan*-FRT	This study	Imaging

SK308	MG1655 *rne::rne-mEos3.2, Δpnp::*FRT-*kan*-FRT	This study	Imaging
SK360	MG1655 *ΔaraFGH araE*::P13-*araE araBAD*::*rne-yfp-kan rne*::*rne-mcherry* FRT-*cat*-FRT	This study	Cloning
SK364	MG1655 *ΔaraFGH araE*::P13-*araE araBAD*::*rne-yfp rne*::*rne-mcherry*	This study	Cloning
SK370	MG1655 Δ(*lacYA*) *rne::rne*(1-592)-*yfp* FRT-*kan*-FRT	(Kim et al., 2024)	qRT-PCR
SK373	MG1655 *rne::rne*(1-529)-*mEos3.2* FRT-*kan*-FRT	This study	Imaging
SK374	MG1655 *rne::rne*(1-592)-*mEos3.2* FRT-*kan*-FRT	This study	Imaging
SK384	MG1655 *rne::rne*(1-592)-*yfp*	This study	Cloning
SK394	MG1655 Δ*lacYA ΔaraFGH araE*::P13-*araE araBAD::rne-yfp rne::rne-mcherry*	This study	qRT-PCR
SK404	MG1655 *rne::rne*(1-564)-*lacY-mEos3.2* FRT-*kan*-FRT	This study	Imaging
SK405	MG1655 Δ*(lacYA) rne::rne*(1-564)-*lacY-mEos3.2* FRT-*kan*-FRT	This study	qRT-PCR
SK407	MG1655 *lacZYA::lacZ-mEos3.2* FRT-*kan*-FRT	This study	Imaging
SK411	MG1655 *rne::rne-mEos3.2*<br><br>pUC19-lacI-lacZonly (amp)	This study	Imaging
SK424	MG1655 *lacYA::lacY*(1-73)-*mEos3.2* FRT-*kan*-FRT	This study	Imaging
SK425	MG1655 *lacYA::lacY*(1-192)-*mEos3.2* FRT-*kan*-FRT	This study	Imaging
SK455	MG1655 *ΔlacIZYA*<br><br>pUC19-lacI-plac-mEos3.2-MTS (amp)	This study	Imaging
SK466	MG1655 *rne::rne*(1-564)-*lacY*(1-73)-*rne*CTD-	This study	Imaging

	mEos3.2 FRT-*kan*-FRT		
SK467	MG1655 *rne::rne*(1-564)-*lacY*(1-192)-*rne*CTD-*mEos3.2* FRT-*kan*-FRT	This study	Imaging
SK482	MG1655 *rne*::*rne-venus hupA*::*hupA-mcherry* FRT-*kan*-FRT	This study	Imaging
SK486	MG1655 *rne*::*rne*(1-592)-*venus hupA*::*hupA-mcherry* FRT-*kan*-FRT	This study	Imaging
SK505	MG1655 Δ(*lacYA*) *rne::rne*(1-564)-*lacY*(1-73)-*rne*CTD-*mEos3.2* FRT-*kan*-FRT	This study	qRT-PCR
SK506	MG1655 Δ(*lacYA*) *rne::rne*(1-564)-*lacY*(1-192)-*rne*CTD-*mEos3.2* FRT-*kan*-FRT	This study	qRT-PCR
SK507	MG1655 *rne::rne*(1-564)-*lacY*(1-73)-*mEos3.2* FRT-*kan*-FRT	This study	Imaging
SK508	MG1655 Δ(*lacYA*) *rne::rne*(1-564)-*lacY*(1-73)-*mEos3.2* FRT-*kan*-FRT	This study	qRT-PCR
SK512	MG1655 *rne::rne-mEos3.2 hupA::hupA-mcherry kan*	This study	Imaging
SK592	MG1655 *rne::rne*(1-564)-*lacY*(1-192)-*mEos3.2* FRT-*kan*-FRT	This study	Imaging
SK593	MG1655 Δ(*lacYA*) *rne::rne*(1-564)-*lacY*(1-192)-*mEos3.2* FRT-*kan*-FRT	This study	qRT-PCR
SK594	MG1655 Δ(*lacYA*) *rne::rne*(1-592)-*yfp*	This study	Cloning
SK595	MG1655 Δ(*lacYA*) *rne::rne-mEos3.2* FRT-*kan*-FRT	This study	qRT-PCR
SK598	MG1655 Δ(*lacYA*) *rne::rne*(1-564)-*lacY-rne*CTD-*mEos3.2* FRT-*kan*-FRT	This study	Imaging<br>qRT-PCR
SK741	MG1655 Δ(*lacYA*) *rne*::*rne*(1-564)-	This study	Imaging

	MTS(F574AF575A)- *rne*CTD -*mEos3.2* FRT-*kan*-FRT		qRT-PCR
SK742	MG1655 Δ(*lacYA*) *rne*::*rne*(1-564)-MTS(F575E)-*rne*CTD-*mEos3.2* FRT-*kan*-FRT	This study	Imaging<br>qRT-PCR
SK743	MG1655 Δ(*lacYA*) *rne*::*rne*(1-564)-MTS(F582E)-*rne*CTD-*mEos3.2* FRT-*kan*-FRT	This study	Imaging<br>qRT-PCR
SK748	MG1655 Δ(*lacYA*) *rne*::*rne*(1-564)-MTS(F574AF575A)-*mEos3.2* FRT-*kan*-FRT	This study	Imaging<br>qRT-PCR
SK749	MG1655 Δ(*lacYA*) *rne*::*rne*(1-564)-MTS(F575E)-*mEos3.2* FRT-*kan*-FRT	This study	Imaging<br>qRT-PCR
SK750	MG1655 Δ(*lacYA*) *rne*::*rne*(1-564)-MTS(F582E)-*mEos3.2* FRT-*kan*-FRT	This study	Imaging<br>qRT-PCR
**Plasmids**			
pBAD18Kan		(Guzman et al., 1995)	Cloning
pET29b-H6_<br>Streptavidin<br>_sfGFP		A gift from Mark Arbing (Addgene plasmid # 124296; http://n2t.net/addgene:124296; RRID: Addgene_124296)	Cloning
pKD13		(Datsenko and Wanner, 2000)	Cloning
pUC19			Cloning
SJK1606	pBAD18kan-mEos3.2-MTS	This study	Cloning
SJK1689	pUC19-lacI-lacY2-CTD-mEos3.2-Kan	This study	Cloning
SJK1697	pUC19-lacI-lacY6-CTD-mEos3.2-frtKanfrt	This study	Cloning

SJK1716	pUC19-lacI-lacY12-CTD-mEos3.2-frtKanfrt	This study	Cloning
SK141	pUC19-lacI-lacZonly	CJW6647 (Kim et al., 2019)	Cloning
SK189	pBAD18 rne-yfp-kan	C. Jacobs-Wagner, JRH515	Cloning
SK567	pET29b-H6_Streptavidin_mEos3.2	This study	Cloning
