# Supplementary material for: Roles of the membrane-binding motif and the C-terminal domain of RNase E in localization and diffusion in E. coli
Source: eLife. 2025 Nov 7;14:RP105062. doi: 10.7554/eLife.105062 (PMC12594526; doi:10.7554/eLife.105062)
Supplement: Supplementary file 2. [file elife-105062-supp2.pdf]

Supplementary file 2. Strain construction

Strain number	Construction
SK187	*mEos3.2-kan* was integrated to the end of *rne* on the chromosome of MG1655 by lambda Red recombination.
SK189	*rne-yfp* sequence was from pVK207 (Khemici et al., 2008).
SK249	*mEos3.2-kan* region in SK187 was amplified using the following primers and integrated into SK107 to replace *mCherry* at the end of *rne*ΔMTS by lambda Red recombination.<br>SJK033: GTGCCGCAGGTGGTCATACG<br>SJK034: GGTTAGCAAGGATGCCATTCG
SK290	The *kan* cassette in SK187 was removed by FLP recombination.
SK292	*mEos3.2-kan* region in SK187 was amplified using the following primers and integrated to the end of *lacY* in MG1655 by lambda Red recombination.<br>K018:<br>GCGGCCCCGGCCCGCTTTCCCTGCTGCGTCGTCAGGTGAATGAAGTCGCTAGA GGTGGTTTATCCATGTCGGCGATCAAGCCGGAC<br>K019:<br>GCTGAACTTGTAGGCCTGATAAGCGCAGCGTATCAGGCAATTTTTATAATTTATC CTTAGTTCCTATTCC
SK304	The *kan* cassette was amplified from pKD13 using the following primers and integrated into the chromosome of SK290 to replace *rhlB* gene by lambda Red recombination.<br>rhlB_KO_F:<br>CGGATACGCTTTCGTAAAGCAATAGTAAGCTGATATTCTACCACACTATGATTCC GGGGATCCGTCGACC<br>rhlB_KO_R:<br>TGAATGATTTTGAGTATGACATTTTTTATTTAACCTGAACGACGACGATTTGTAG GCTGGAGCTGCTTCG
SK308	The *kan* cassette was amplified from pKD13 using the following primers and integrated into the chromosome of SK290 to replace *pnp* gene by lambda Red recombination.<br>pnp_KO_F:<br>CCCGCCGCAGCGGAGGGCAAATGGCAACCTTACTCGCCCTGTTCAGCAGCATT CCGGGGATCCGTCGACC<br>pnp_KO_R:<br>ACACCAGTGCCGTAAGGTACTGTCTAAGAAAGAGAAAGGATATTACATTGTGTA GGCTGGAGCTGCTTCG
SK360	First, *rne-mcherry-cat* in SK72 was moved to SK52 via phage transduction (SK349). Next, *rne-yfp-kan* was amplified from SK189 using primer K050 and K053 and integrated into *araBAD* locus on the chromosome of SK349 by lambda Red recombination.<br>K050:<br>GCAACTCTCTACTGTTTCTCCATACCCGTTTTTTTGGATGGAGTGAAACGATGAA AAGAATGTTAAT<br>K053:

	GCTTGAGTATAGCCTGGTTTCGTTTGATTGGCTGTGGTTTTATACAGTCAAAGTA TATATGAGTAAACTTGG
SK364	From SK360, both *kan* and *cat* cassettes were removed by FLP recombination.
SK373	*mEos3.2-kan* region in SK187 was amplified using the primers K066 and SJK034 and then integrated into the *rne* region in MG1655 by lambda Red recombination.<br>K066:<br>CGTCTGAAGAAGAGTTCGCTGAACGTAAGCGTCCGGAACAACCTGCGCTGCTC GAGGGTCCGGCTGGTCTGATGTCG
SK374	*mEos3.2-kan* region in SK187 was amplified using the primers K065 and SJK034 and then integrated into MG1655 by lambda Red recombination.<br>K065:<br>GCGCACTGAAAGCGCTGTTCAGCGGTGGTGAAGAAACCAAACCGACCGAGCTC GAGGGTCCGGCTGGTCTGATGTCG
SK384	The *kan* cassette in SK186 was removed by FLP.
SK394	To make a clean *lacYA* deletion, *cat-sacB* from pEL04 was integrated into *lacYA* region in SK364 and then replaced by synthetic DNA lacZAfor and its complementary lacZArev.<br>lacZAfor:<br>AGCTGAGCGCCGGTCGCTACCATTACCAGTTGGTCTGGTGTCAAAAATAAATTA TAAAAATTGCCTGATACGCTGCGCTTATCAGGCCTACAAGTTCAGC
SK404	*lacY-mEos3.2-kan* region was amplified from SK292 using primer K088 and K089 and used to replace the second half of rne in the chromosome of MG1655 by lambda Red recombination.<br>K088:<br>CGCCTGTTGTAGCTCCAGCACCGAAAGCTGCACCGGCAACACCAGCAGCTTAC TATTTAAAAAACACAAACTTTTGG<br>K089:<br>AATAAAAAAAGCCCTGGCAGTTACCAGGGCTTGATTACTTTGAGCTAATTATTATC CTTAGTTCCTATTCC
SK405	Same as SK404, but the DNA fragment was integrated into SK98.
SK407	*mEos3.2-kan* region was amplified from SK292 using primer K098 and lacA_out50R and integrated into the end of *lacZ* in MG1655 by lambda Red recombination.<br>K098:<br>TCCAGCTGAGCGCCGGTCGCTACCATTACCAGTTGGTCTGGTGTCAAAAAAGA GGTGGTTTATCCATGTCG<br>lacA_out50R: GCTGAACTTGTAGGCCTGATAAGC
SK411	SK141 plasmid was electroporated into SK290.
SK424	*mEos3.2-kan* was amplified from SK187 using primer K099 and K028 and integrated into the *lacY* region in MG1655 by lambda Red recombination.<br>K099:<br>TATTCCAACCGCTGTTTGGTCTGCTTTCTGACAAACTCGGGCTGCGCAAAAGAG GTGGTTTATCCATGTCGGCGATCAAGCCGGAC<br>K028:<br>TGTAGATCGCTGAACTTGTAGGCCTGATAAGCGCAGCGTATCAGGCAATTTATC GTGAGGATGCGTCATCG

SK425	Similar to SK424, but K101 and K028 primers were used to prepare the DNA fragment.<br>K101:<br>CACTCATCCTCGCCGTTTTACTCTTTTTCGCCAAAACGGATGCGCCCTCTAGAG GTGGTTTATCCATGTCGGCGATCAAGCCGGAC
SK455	The plasmid was made by Gibson ligation of two fragments: (1) pUC19 backbone and *lacI* region of plasmid SK141 using two primers: lacZp_rev and lacA_out20F and (2) *mEos3.2*-MTS from SJK1606 (3'MTS). Here, MTS is a 51 base sequence from *rne*, and *mEos3.2* sequence is fused at the 5' side. The resulting plasmid was electroporated into SK105.<br>lacZp_rev: CATAGCTGTTTCCTGTGTGAAATTGTTATCC<br>lacA_out20F: ATTATAAAAATTGCCTGATACG
SK466	*lacY*-CTD-*mEos3.2-kan* was amplified from plasmid SJK1689 using K088 and K089 and integrated into the *rne* region in SK384 by lambda Red recombination.
SK467	Same as SK466 but plasmid SJK1697 was used.
SK482	This strain was constructed in two steps. First, we constructed *rne*::*rne-venus* by integrating *venus* into the *rne* region in MG1655 by lambda Red recombination. The *kan* cassette was removed by FLP.<br><br>rne-venus-F:<br>CGGCAACACATCATGCCTCTGCCGCTCCTGCGCGTCCGCAACCTGTTGAGAGA GGTGGTTTATCCAGCAAGG<br>rne-venus-R:<br>AATAAAAAAGCCCTGGCAGTTACCAGGGCTTGATTACTTTGAGCTAATTATCGCT GGTGTAGGCTGGAGC<br><br>Secondly, phage transduction was performed to move *hupA*::*hupA-mCherry-kan* (SK213) into this strain.
SK486	This strain was constructed in two steps, similar to SK482. Only difference is that rne592-Venus-F was used to amplify *venus* when we constructed *rne*::*rne*(1-592)-*venus*.
SK505	Phage transduction of *rne* mutant in SK466 into SK98.
SK506	Phage transduction of *rne* mutant in SK467 into SK98.
SK507	*lacY2-mEos3.2-kan* region was from SK424 using K088 and K140 and inserted into *rne* sequence in MG1655.<br>K140:<br>AATAAAAAAGCCCTGGCAGTTACCAGGGCTTGATTACTTTGAGCTAATTATATCG TGAGGATGCGTCATCG
SK508	Same as SK507 except that the DNA was integrated into SK98 for lambda Red recombination.
SK512	*hupA-mcherry-kan* in CJW5158 was moved to SK290 via phage transduction.
SK592	*lacY6-mEos3.2-kan* region was from SK425 using K088 and K140 and inserted into *rne* sequence in MG1655.

SK593	Same as SK592, but the DNA was integrated into SK98 by lambda Red recombination.
SK594	The *kan* cassette was removed from SK370 by FLP.
SK595	Phage transduction of SK187 into SK98.
SK598	Same as SK466, but plasmid SJK1716 was used for PCR, and the integration occurred into SK594.
SK741	The DNA sequence encoding the mutant MTS (F574AA)-CTD-mEos3.2-Kan was amplified from SK187 using the primers F574AA_for and SJK034. The amplicon was integrated into SK594 by lambda Red recombination.<br>F574AA_for:<br>CTGCACCGGCAACACCAGCAGCTCCTGCACAACCTGGGCTGTTGAGCCGCGCAGCAGGCGCACTGAAAGCGCTGTTCAGC
SK742	The DNA sequence encoding the mutant MTS (F575E)-CTD-mEos3.2-Kan was amplified from SK187 using the primers F575E_for and SJK034. The amplicon was integrated into SK594 by lambda Red recombination.<br>F575E_for:<br>CACCGGCAACACCAGCAGCTCCTGCACAACCTGGGCTGTTGAGCCGCTTCGAAGGCGCACTGAAAGCGCTGTTCAGC
SK743	The DNA sequence encoding the mutant MTS (F582E)-CTD-mEos3.2-Kan was amplified from SK187 using the primers F582E_for and SJK034. The amplicon was integrated into SK594 by lambda Red recombination.<br>F582E_for:<br>CTGCACAACCTGGGCTGTTGAGCCGCTTCTTCGGCGCACTGAAAGCGCTGGAAAGCGGTGGTGAAGAAACCAAACC
SK748	The DNA sequence encoding the mutant MTS (F574AA)-mEos3.2-Kan was amplified from the DNA fragment used to construct SK374. It was amplified using the primers F574AA_for and SJK034. The amplicon was integrated into SK98 by lambda Red recombination.
SK749	The DNA sequence encoding the mutant MTS (F575E)-mEos3.2-Kan was amplified from the DNA fragment used to construct SK374. It was amplified using the primers F575E_for and SJK034. The amplicon was integrated into SK98 by lambda Red recombination.
SK750	The DNA sequence encoding the mutant MTS (F582E)-mEos3.2-Kan was amplified from the DNA fragment used to construct SK374. It was amplified using the primers F582E_for and SJK034. The amplicon was integrated into SK98 by lambda Red recombination.
**Plasmids**	
SJK1606	We constructed pBAD18kan-Venus-MTS (SJK1591) first by Gibson ligation of 3 DNA fragments. Two fragments were from the plasmid backbone (pBAD18kan (Guzman et al., 1995)), amplified by K042 and aph_in330F and by K061 and aph_in355rev. K061 primer contains the MTS sequence. The third DNA fragment was *venus* sequence amplified from SX701 (Choi et al., 2008) using K046 and K062. K046 contains a proper RBS sequence for translation of *venus*-MTS from the final plasmid. K062 contains a linker sequence between Venus and the MTS.

	The second Gibson ligation was done with two DNA fragments: linearized plasmid SJK1591 by PCR with K056 and K057 and *mEos3.2* sequence from SK187 by PCR with mEos_5for and mEos_3rev.<br><br>K042: CTAGCCCAAAAAAACGGGTATGG<br>Aph_in330F: CCAGGTATTAGAAGAATATCC<br>K061:<br>CAACCTGGGCTGTTGAGCCGCTTCTTCGGCGCACTGAAAGCGCTGTTCAGCTA<br>ACCTGATACAGATTAAATCAGAACG<br>aph_in355rev: CTGAATCAGGATATTCTTCTAATACC<br>K046:<br>CCATACCCGTTTTTTTGGGCTAGTTGGATGGAGTGAAACGATGAGCAAGGGCG<br>AGGAGCTGTTCACC<br>K062:<br>GCCGAAGAAGCGGCTCAACAGCCCAGGTTGGGATAAACCACCTCTTAGCC<br>K056:<br>CACTCGGGCCTGCCGGACAACGCCCGCCGCAAGGGTGGGCGCGCCGACCC<br>K057:<br>GATCTTCATGTCCGGCTTGATCGCCGACATCGTTTCACTCCATCCAACTAGC<br>mEos_5for: ATGTCGGCGATCAAGCCGGAC<br>mEos_3rev: GCGGCGGGCGTTGTCCGGCAG
SJK1689	The plasmid was constructed by Gibson ligation of 3 DNA fragments. The first fragment is pUC19 plasmid backbone and *lacI* sequence amplified from plasmid SK141 using two primers: lacZp_rev and lacA_out20F. The second fragment is *lacY2* sequence amplified from MG1655 using primers K117 and K118. The third fragment is CTD-*mEos3.2-kan* sequence amplified from SK187 using rne_in1755F and K124.<br>K117:<br>GATAACAATTTCACACAGGAAACAGCTATGTACTATTTAAAAAACACAAACTTTT<br>GG<br>K118:<br>TGCTGGTTGCTCGGTCGGTTTGGTTTCTTCTTTGCGCAGCCCGAGTTTGTCAGA<br>AAGC<br>rne_in1755F: GAAGAAACCAAACCGACCGAGC<br>K124:<br>GATAAGCGCAGCGTATCAGGCAATTTTTATAATATAAAAAAGCCCTGGCAGTTAC<br>C
SJK1697	The plasmid was constructed in the same way as for SJK1689 except for a different second fragment. It was *lacY6* sequence amplified from MG1655 using primers K117 and K120.<br>K120:<br>TGCTGGTTGCTCGGTCGGTTTGGTTTCTTCAGAAGAGGGCGCATCCGTTTTGG
SJK1716	The plasmid was constructed in the same way as for SJK1689 except for a different second fragment. It was *lacY12* sequence amplified from MG1655 using primers K117 and K123.<br>K123:<br>TGCTGGTTGCTCGGTCGGTTTGGTTTCTTCAGCGACTTCATTCACCTGACG
SK567	The plasmid was constructed by Gibson ligation of two fragments. The first fragment was from pET29b-H6_Streptavidin_sfGFP by digestion with BamHI. The second fragment was *mEos3.2* sequence, amplified from SK187 using primers strep-mEOS-f and strep-mEOS-r.

	strep-mEOS-f:<br>GAATCCGTTGGACGCTGTCCAACAAGGATC**G**GGATCCGGATCAATGTCGGCGA<br>TCAAGCCGGACATGAAGATCAAGC<br>strep-mEOS-r:<br>GTTCTTCTCCTTTGCTCATTGATCCGGATCCTTAGCGGCGGGCGTTGTCC
