# Supplementary material for: Roles of the membrane-binding motif and the C-terminal domain of RNase E in localization and diffusion in E. coli
Source: eLife. 2025 Nov 7;14:RP105062. doi: 10.7554/eLife.105062 (PMC12594526; doi:10.7554/eLife.105062)
Supplement: Supplementary file 3. [file elife-105062-supp3.pdf]

## Supplementary file 3. Doubling times and cell sizes

| Strain Number | Doubling time (min, mean ± std) | Cell length (µm, mean ± std) | Cell width (µm, mean ± std) |
|---|---|---|---|
| SK1 (MG1655) | 82 ± 10 | – | – |
| SK98 | 95.0 ± 9.40 | -- | -- |
| SK187 | 89 ± 9 | 3.362 ± 0.475 | 1.068 ±0.066 |
| SK249 | 106 ± 6 | 3.582 ± 0.547 | 1.053 ±0.067 |
| SK292 | 83.2 ± 3.2 | 3.287 ± 0.479 | 1.088 ±0.050 |
| SK304 | 93 ± 11 | 3.284 ± 0.516 | 1.083 ±0.054 |
| SK308 | 69.8 ± 1.1 | 3.206 ± 0.479 | 1.083 ±0.050 |
| SK373 | 78 ± 4 | 3.459 ± 0.447 | 1.122 ±0.063 |
| SK374 | 70.8 ± 3.7 | 3.333 ± 0.444 | 1.139 ±0.057 |
| SK404 | 90 ± 7 | 3.27 ± 0.03 | 1.161 ± 0.004 |
| SK411 | 87.6 ± 3.6 | 3.26 ± 0.03 | 1.121 ± 0.004 |
| SK424 | 83 ± 8 | 3.24 ± 0.03 | 1.103 ± 0.004 |
| SK425 | 81 ± 7 | 3.35 ± 0.02 | 1.098 ± 0.003 |
| SK455 | 88 ± 2 | 3.48 ± 0.03 | 1.113 ± 0.002 |
| SK466 | 87 ± 11 | 3.18 ± 0.03 | 1.119 ± 0.003 |
| SK467 | 89 ± 10 | 3.25 ± 0.03 | 1.093 ± 0.004 |
| SK507 | 89 ± 10 | 3.09 ± 0.03 | 1.142 ± 0.005 |
| SK592 | 94.1 ± 0.9 | 3.27 ± 0.02 | 1.188 ± 0.003 |
| SK595 | 98.5 ± 3.3 | -- | -- |
| SK598 | 93.6 ± 7.2 | 3.08 ± 0.02 | 1.050 ± 0.002 |
| SK741 | 94.0 ± 3.6 | 3.49 ± 0.03 | 1.034 ± 0.002 |
| SK742 | 99.7 ± 4.6 | 3.43 ± 0.03 | 1.031 ± 0.003 |
| SK743 | 102.4 ± 3.0 | 3.51 ± 0.03 | 1.047 ± 0.002 |
| SK748 | 94.8 ± 4.0 | 3.59 ± 0.03 | 1.203 ± 0.003 |
| SK749 | 95.2 ± 3.4 | 3.34 ± 0.03 | 1.183 ± 0.004 |

| | | | |
|---|---|---|---|
| SK750 | 93.7 ± 3.3 | 3.33 ± 0.02 | 1.161 ± 0.003 |
| SK187<br>(M9 succinate at 30°C) | 153 ± 19 min | 3.37 ± 0.02 | 0.970 ± 0.002 |
