# Supplementary material for: Roles of the membrane-binding motif and the C-terminal domain of RNase E in localization and diffusion in E. coli
Source: eLife. 2025 Nov 7;14:RP105062. doi: 10.7554/eLife.105062 (PMC12594526; doi:10.7554/eLife.105062)
Supplement: Supplementary file 4. [file elife-105062-supp4.pdf]

Supplementary file 4. xNorm histogram modeling results

Protein	Strain number	fcut		locError (nm)		dilF		MB%	
		mean	std	mean	std	mean	std	mean	std
RNE	SK187	0.3	0.030	58.4	1.27	1.67	0.010	92.6	1.2
LacY	SK292	0.25	0.006	49.3	0.52	1.62	0.002	98.5	1.0
LacZ	SK407	0.36	0.020	56.3	0.92	1.87	0.020	3.4	2.1
RNE-F574AA -CTD	SK741	0.26	0.01	55.0	0.56	1.76	0.004	88.12	1.71
RNE-F582E-CTD	SK743	0.28	0.01	49.1	1.05	1.9	0.002	46.84	1.43
RNE-F575E-CTD	SK742	0.27	0.01	49.8	1.01	1.9	0.002	49.71	1.48
RNE ΔMTS	SK249	0.29	0.01	45.4	1.26	1.9	0.003	33.71	1.33
MTS	SK455	0.28	0.006	47.5	0.55	1.52	0.003	99.9	0.1
LacY2	SK424	0.26	0.005	48.3	0.68	1.58	0.003	99.6	0.4
LacY6	SK425	0.26	0.006	48.1	0.47	1.57	0.002	90.2	0.6
RNE ΔCTD or RNE (1-592)	SK374	0.27	0.004	52.4	0.52	1.56	0.002	99.8	0.2
RNE-LacY2 (ΔCTD)	SK507	0.31	0.008	49.9	0.8	1.56	0.004	99.8	0.2
RNE-LacY6 (ΔCTD)	SK592	0.3	0.008	45.6	0.91	1.49	0.004	99.8	0.2
RNE-LacY12 (ΔCTD)	SK404	0.27	0.006	57.1	0.6	1.55	0.002	99.3	0.6
RNE-LacY2-CTD	SK466	0.27	0.020	55	0.64	1.7	0.004	69.4	1.9
RNE-LacY6-CTD	SK467	0.29	0.020	56.1	0.85	1.72	0.007	86.2	1.5
RNE-LacY12-CTD	SK598	0.36	0.020	53.7	0.81	1.73	0.010	97.1	1.8
RNE in M9succ	SK187	0.35	0.030	59	0.87	1.86	0.020	92.1	2.7
RNE-LacY2-CTD in M9succ	SK466	0.25	0.007	48	1.18	1.67	0.009	43.1	1.2
MTS in M9succ	SK455	0.32	0.040	59.8	0.92	1.67	0.020	82.8	3.4
LacY2 in M9succ	SK424	0.29	0.006	46.7	0.49	1.7	0.004	99.9	0.1
RNE-F574AA (ΔCTD)	SK748	0.31	0.0062	50.2	0.63	1.54	0.003	96	0.56
RNE-F582E (ΔCTD)	SK750	0.24	0.0042	54.7	0.54	1.59	0.002	67.1	0.77
RNE-F575E (ΔCTD)	SK749	0.27	0.0070	52.8	0.48	1.55	0.002	91.4	0.37
RNE ΔMTS ΔCTD or RNE (1-529)	SK373	0.33	0.02	59.6	0.59	1.89	0.005	17.4	0.99
