# Supplementary material for: Roles of the membrane-binding motif and the C-terminal domain of RNase E in localization and diffusion in E. coli
Source: eLife. 2025 Nov 7;14:RP105062. doi: 10.7554/eLife.105062 (PMC12594526; doi:10.7554/eLife.105062)
Supplement: Supplementary file 5. [file elife-105062-supp5.pdf]

Supplementary file 5. qRT PCR primers used in study

Description	Primer sequence (5' to 3')
lacZ530F	TTTTACGCGCCGGAGAAAAC
lacZ530R	AGTCGGTTTATGCAGCAACG
lacZ2732F	TTACTGCCGCCTGTTTTGAC
lacZ2732R	TGTAGCGGCTGATGTTGAAC
gapA274F	GTTGTCGCTGAAGCAACTGG
gapA274R	CGATGTCCTGGCCAGCATAT
