# Supplementary material for: Roles of the membrane-binding motif and the C-terminal domain of RNase E in localization and diffusion in E. coli
Source: eLife. 2025 Nov 7;14:RP105062. doi: 10.7554/eLife.105062 (PMC12594526; doi:10.7554/eLife.105062)
Supplement: Supplementary file 6. [file elife-105062-supp6.pdf]

Supplementary file 6. Figure data statistics

Figure number	Strain	Strain	Number of tracks/spots	Number of cells
1D	SK187	RNE	143,700 spots	179
	SK292	LacY	199,228 spots	161
1F	SK407	LacZ	218,120 spots	247
1-SF1	SK187	RNE	143,700spots	179
1-SF2A,B	SK187, live	WT RNE, live		179
	SK187, fixed	WT RNE, fixed		249
	SK249, live	RNE ΔMTS, live		96
	SK249, fixed	RNE ΔMTS, fixed		282
2B	SK407	LacZ	218,120 spots	247
	SK249	RNE ΔMTS	91,960 spots	96
	SK373	RNE ΔMTS ΔCTD	583,156 spots	205
2D	SK187	WT RNE	143,700 spots	179
	SK741	RNE-F574AA-CTD	254,252 spots	417
	SK743	RNE-F582E-CTD	284,540 spots	449
	SK742	RNE-F575E-CTD	222,496 spots	354
	SK249	RNE ΔMTS	91,960 spots	96
3A	SK187	RNE, EATA MSD	11,260 tracks	177
3B	SK187	WT RNE	11,260 tracks	177
	SK249	RNE ΔMTS	7,539 tracks	95
	SK374	RNE ΔCTD	37,739 tracks	215
	SK373	RNE ΔMTS ΔCTD	36,858 tracks	205
3C	SK187	RNE	11,260 tracks	179
	SK187 +rif	RNE +rif	7,473 tracks	280
3D	SK292	LacY	20,186 tracks	159
	SK292 +rif	LacY +rif	3,364 tracks	140
3E	SK47	L1	2,533 tracks	74
	SK47 +rif	L1 +rif	1,109 tracks	51
3-SF1	SK187	WT RNE	11,260 tracks	177
3-SF2	SK187	RNE	11,260 tracks	177
	SK187 +rif	RNE +rif	7,473 tracks	280
	SK47	L1 ribosome	2,533 tracks	74
	Protein extracted from plasmid SK567	His6-streptavidin-mEos3.2	10,291 tracks	--
3-SF3	SK47	L1 ribosome	2,533 tracks	74
3-SF4C	SK187	WT RNE	11,260 tracks	177
	SK187 +chlor	RNE +chlor	1,887 tracks	52
	SK411	RNE, *lacZ* overexpressed	15,630 tracks	279
4B	SK455	MTS	224,352 spots	577
	SK424	LacY2	107,436 spots	216
	SK425	LacY6	221,840 spots	367
	SK292	LacY12 (full)	199,228 spots	161
4C	SK455	MTS	18,804 tracks	558
	SK424	LacY2	3,812 tracks	175
	SK425	LacY6	16,009 tracks	336
	SK292	LacY12	20,186 tracks	159

5C	SK292	LacY	199,228 spots	161
	SK374	RNE ΔCTD	371,680 spots	215
	SK507	RNE-LacY2 ΔCTD	72,872 spots	323
	SK592	RNE-LacY6 ΔCTD	242,556 spots	396
	SK404	RNE-LacY12 ΔCTD	189,584 spots	141
5D	SK292	LacY	199,228 spots	161
	SK187	WT RNE	143,700 spots	179
	SK466	RNE-LacY2-CTD	218,416 spots	250
	SK467	RNE-LacY6-CTD	189,672 spots	180
	SK598	RNE-LacY12-CTD	105,656 spots	450
5G	SK374	RNE ΔCTD	37,739 tracks	215
	SK507	RNE-LacY2 ΔCTD	4,431 tracks	273
	SK592	RNE-LacY6 ΔCTD	11,375 tracks	371
	SK404	RNE-LacY12 ΔCTD	20,263 tracks	140
5H	SK187	WT RNE	11,260 tracks	177
	SK466	RNE-LacY2-CTD	17,698 tracks	247
	SK467	RNE-LacY6-CTD	11,323 tracks	176
	SK598	RNE-LacY12-CTD	8,060 tracks	402
5-F2F	SK486, fixed	RNE ΔCTD, fixed		133
	SK482, fixed	WT RNE, fixed		123
A1-F1A	SK187	WT RNE	143,700 spots-all<br>34,040 spots-slow<br>2,376 spots-fast	179
A1-F1B	SK292	LacY	199,228 spots-all<br>60,984 spots-slow<br>888 spots-fast	161
A1-F1C	SK407	LacZ	218,120 spots-all<br>860 spots-slow<br>25,548 spots-fast	247
A1-F1D	SK249	RNE ΔMTS	91,960 spots-all<br>8,720 spots-slow<br>17,816 spots-fast	96
A1-F1E	SK373	RNE ΔMTS ΔCTD	583,156 spots-all<br>21,432 spots-slow<br>104,228 spots-fast	205
A1-F1F	SK741	RNE-F574AA-CTD	254,252 spots-all<br>66,832 spots-slow<br>8,700 spots-fast	417
A1-F1G	SK743	RNE-F582E-CTD	284,540 spots-all<br>23,748 spots-slow<br>42,528 spots-fast	449
A1-F1H	SK742	RNE-F575E-CTD	222,496 spots-all<br>27,588 spots-slow<br>27,420 spots-fast	354
A1-F1I	SK425	LacY6	221,840 spots-all<br>42,388 spots-slow<br>4,960 spots-fast	367
A1-F1J	SK466	RNE-LacY2-CTD	218,416 spots<br>39,276 spots-slow<br>17,904 spots-fast	250
A1-F1K	SK467	RNE-LacY6-CTD	189,672 spots-all<br>32,508 spots-slow<br>5,100 spots-fast	180

A1-F1L	SK598	RNE-LacY12-CTD	105,656 spots-all<br>24,308 spots-slow<br>696 spots-fast	450
A1-F1M	SK748	RNE-F574AA ΔCTD	541,224 spots-all<br>134,388 spots-slow<br>5,508 spots-fast	350
A1-F1N	SK750	RNE-F582E ΔCTD	489,076 spots-all<br>67,636 spots-slow<br>32,852 spots-fast	435
A1-F1O	SK749	RNE-F575E ΔCTD	259,736 spots-all<br>59,900 spots-slow<br>5,920 spots-fast	228

Abbreviations:

SF supplement figure
A1-F Appendix 1-figure
