# Supplementary material for: Roles of the membrane-binding motif and the C-terminal domain of RNase E in localization and diffusion in E. coli
Source: eLife. 2025 Nov 7;14:RP105062. doi: 10.7554/eLife.105062 (PMC12594526; doi:10.7554/eLife.105062)
Supplement: Supplementary file 7. [file elife-105062-supp7.pdf]

Supplementary file 7. *P*-values determined by two-tailed Student's *t*-test

Relevant figure	Alternative hypothesis	*p*-value
6C	k_{d1} of *lacZ* mRNA in RNE-F574AA-CTD is different from that in WT RNE.	0.66
	k_{d1} of *lacZ* mRNA in RNE-F575E-CTD is different from that in WT RNE.	3.8E-04
	k_{d1} of *lacZ* mRNA in RNE-F582E-CTD is different from that in WT RNE.	0.0076
	k_{d1} of *lacZ* mRNA in RNE-LacY2-CTD is different from that in WT RNE.	0.021
	k_{d1} of *lacZ* mRNA in RNE-LacY6-CTD is different from that in WT RNE.	0.60
	k_{d1} of *lacZ* mRNA in RNE-LacY12-CTD is different from that in WT RNE.	0.41
	k_{d1} of *lacZ* mRNA in RNE ΔMTS is different from that in WT RNE.	0.013
6D	k_{d2} of *lacZ* mRNA in RNE-F574AA-CTD is different from that in WT RNE.	0.12
	k_{d2} of *lacZ* mRNA in RNE-F575E-CTD is different from that in WT RNE.	0.97
	k_{d2} of *lacZ* mRNA in RNE-F582E-CTD is different from that in WT RNE.	0.44
	k_{d2} of *lacZ* mRNA in RNE-LacY2-CTD is different from that in WT RNE.	0.67
	k_{d2} of *lacZ* mRNA in RNE-LacY6-CTD is different from that in WT RNE.	0.47
	k_{d2} of *lacZ* mRNA in RNE-LacY12-CTD is different from that in WT RNE.	0.81
	k_{d2} of *lacZ* mRNA in RNE ΔMTS is different from that in WT RNE.	0.23
6E	k_{d1} of *lacZ* mRNA in RNE-F574AA ΔCTD is different from that in RNE ΔCTD.	0.50
	k_{d1} of *lacZ* mRNA in RNE-F575E ΔCTD is different from that in RNE ΔCTD.	0.041
	k_{d1} of *lacZ* mRNA in RNE-F582E ΔCTD is different from that in RNE ΔCTD.	0.031
	k_{d1} of *lacZ* mRNA in RNE-LacY2 ΔCTD is different from that in RNE ΔCTD.	0.58
	k_{d1} of *lacZ* mRNA in RNE-LacY6 ΔCTD is different from that in RNE ΔCTD.	0.37
	k_{d1} of *lacZ* mRNA in RNE-LacY12 ΔCTD is different from that in RNE ΔCTD.	0.37
	k_{d1} of *lacZ* mRNA in RNE ΔMTS ΔCTD is different from that in RNE ΔCTD.	0.034
6F	k_{d2} of *lacZ* mRNA in RNE-F574AA ΔCTD is different from that in RNE ΔCTD.	0.18
	k_{d2} of *lacZ* mRNA in RNE-F575E ΔCTD is different from that in RNE ΔCTD.	0.019
	k_{d2} of *lacZ* mRNA in RNE-F582E ΔCTD is different from that in RNE ΔCTD.	0.020
	k_{d2} of *lacZ* mRNA in RNE-LacY2 ΔCTD is different from that in RNE ΔCTD.	0.27
	k_{d2} of *lacZ* mRNA in RNE-LacY6 ΔCTD is different from that in RNE ΔCTD.	0.14
	k_{d2} of *lacZ* mRNA in RNE-LacY12 ΔCTD is different from that in RNE ΔCTD.	0.0037
	k_{d2} of *lacZ* mRNA in RNE ΔMTS ΔCTD is different from that in RNE ΔCTD.	0.030
6-SF1	k_{d1} of *lacZ* mRNA in RNase E without mEos3.2 attached and k_{d1} of *lacZ* mRNA in RNase E with mEos3.2 attached are different	0.9104
	k_{d2} of *lacZ* mRNA when RNase E was without mEos3.2 attached and k_{d2} of *lacZ* mRNA when RNase E has mEos3.2 attached are different	0.8418
6-SF2	k_{d1} of *lacZ* mRNA in WT and k_{d1} of *lacZ* mRNA when RNase E is overexpressed are different	0.9635

	k_{d2} of *lacZ* mRNA in WT is smaller than k_{d2} of *lacZ* mRNA when RNase E is overexpressed	0.2812

Abbreviations:

SF supplement figure
