# Supplementary material for: Roles of the membrane-binding motif and the C-terminal domain of RNase E in localization and diffusion in E. coli
Source: eLife. 2025 Nov 7;14:RP105062. doi: 10.7554/eLife.105062 (PMC12594526; doi:10.7554/eLife.105062)
Supplement: Supplementary file 8. [file elife-105062-supp8.pdf]

Supplementary file 8. Diffusion coefficient (*D*) and 95% CI

Strain	Strain	<D>	SEM	<D> from bootstrapping	95% CI from bootstrapping
SK47	Ribosome L1	0.053	0.002	0.0528	[0.0525, 0.0530]
SK47 +rif	Ribosome L1 +rif	0.37	0.008	0.372	[0.371, 0.373]
SK187	WT RNE	0.018	0.0002	0.01836	[0.01834, 0.01838]
SK187 +chlor	RNE +chlor	0.019	0.0005	0.01902	[0.01895, 0.01908]
SK187 +rif	RNE +rif	0.027	0.0003	0.02691	[0.02687, 0.02694]
SK187, M9succ	RNE, M9succ	0.038	0.0002	0.03786	[0.03783, 0.03790]
SK249	RNE ΔMTS	0.10	0.001	0.1007	[0.1005, 0.1008]
SK292	LacY	0.078	0.0004	0.07774	[0.07768, 0.07780]
SK292 +rif	LacY +rif	0.097	0.001	0.0973	[0.0972, 0.0975]
SK292, M9succ	LacY, M9succ	0.087	0.0003	0.08702	[0.08698, 0.08707]
SK373	RNE ΔMTS ΔCTD	0.39	0.001	0.3919	[0.3917, 0.3921]
SK374	RNE ΔCTD	0.074	0.0003	0.07403	[0.07398, 0.07408]
SK374, M9succ	RNE592, M9succ	0.038	0.0002	0.03785	[0.03783, 0.03787]
SK404	RNE-lacY ΔCTD	0.049	0.0004	0.04929	[0.04924, 0.04934]
SK407	LacZ	0.43	0.003	0.4322	[0.4317, 0.4326]
SK407, M9succ	LacZ, M9succ	0.38	0.005	0.3783	[0.3777, 0.3790]
SK424	LacY2	0.17	0.002	0.1694	[0.1691, 0.1698]
SK425	LacY6	0.14	0.0009	0.1367	[0.1366, 0.1368]
SK455	MTS	0.11	0.0006	0.10627	[0.10618, 0.10636]
SK466	RNE-LacY2-CTD	0.061	0.0007	0.06107	[0.06098, 0.06116]
SK467	RNE-LacY6-CTD	0.021	0.0002	0.02094	[0.02092, 0.02097]
SK507	RNE-LacY2 ΔCTD	0.098	0.002	0.0987	[0.0984, 0.0989]
SK592	RNE-LacY6 ΔCTD	0.055	0.0005	0.05463	[0.05457, 0.05469]
SK598	RNE-LacY12-CTD	0.017	0.0002	0.01714	[0.01712, 0.01717]
SK741	RNE-F574AA-CTD	0.036	0.0003	0.03645	[0.03640, 0.03650]
SK742	RNE-F575E-CTD	0.094	0.0007	0.09424	[0.09415, 0.09433]
SK743	RNE-F582E-CTD	0.091	0.0006	0.09135	[0.09127, 0.09143]
SK748	RNE-F574AA ΔCTD	0.088	0.0004	0.08781	[0.08775, 0.08788]
SK749	RNE-F575E ΔCTD	0.098	0.0007	0.09815	[0.09805, 0.09825]
SK750	RNE-F582E ΔCTD	0.19	0.001	0.1944	[0.1943, 0.1945]
